# How Expectations and Trust in Telemedicine Contribute to Older Adults’ Sense of Control: An Empirical Study

**DOI:** 10.3390/healthcare12171685

**Published:** 2024-08-23

**Authors:** Siyu Niu, Wenjia Hong, Yiming Ma

**Affiliations:** 1 School of Health Management, Anhui Medical University, Hefei 230032, China; siyuniu198@163.com; 2 School of Management, Hefei University of Technology, Hefei 230009, China; 3 School of Management Science and Engineering, Anhui University of Finance and Economics, Bengbu 233030, China

**Keywords:** expectation, trust, telemedicine, elderly, sense of control in life

## Abstract

As numerous nations transition into digital and aging societies, the digital divide has emerged as a significant impediment to older adults’ autonomous engagement in the digital society. Enhancing the well-being of elderly individuals through remote medical technology represents a prevailing and prospective trend. Nevertheless, remote medical technology extends beyond the realm of healthcare, offering promise for narrowing the digital divide through the deployment of digital devices and provision of intergenerational support. Therefore, this study investigates the role of trust and expectations in the use of telemedicine, indicating potential pathways for how these products can improve older adults’ daily living abilities. Through the construction of a theoretical model, we collected the relevant data of 661 elderly people who use telemedicine technology in China and analyzed the data with SmartPLS4 to obtain the research results. The study discovered that, among older people using telemedicine technology, (1) healthcare expectations promote the breadth of telemedicine product use; (2) trust in product safety increases the depth of telemedicine product use; (3) trust in the service provider promotes the breadth of telemedicine product use; and (4) when compared to the depth of product use, the breadth of telemedicine product use increases older adults’ sense of control over their digital lives. The findings provide new empirical data to support growing beliefs about how expectations and trust can increase a sense of control over one’s life. They also provide practical contributions on how to boost older adults’ usage of telemedicine products, promote their digital literacy and competency, and enhance their sense of control over their digital lives.

## 1. Introduction

The increasing number of older adults in the world puts more pressure on healthcare systems because of many factors, such as an increase in noncommunicable chronic diseases and the increased burden of disease due to multimorbidity and geriatric syndromes (e.g., frailty and dementia) [1]. These factors increase pressure on healthcare use and stretch healthcare services even further than an increase in population would [2]. This has resulted in rising healthcare expenses in countries as their populations reach older ages, a shortage of healthcare providers, and higher household expenditures on healthcare. Therefore, how to expand the coverage of healthcare services and improve the quality of related healthcare services has become an important issue for all countries as they deal with aging populations. According to the World Health Organization, in the information age, harnessing the power of digital technologies is critical to achieving universal health coverage, as they are essential tools to promote health, keep the world safe, and serve the vulnerable [3].

To address the challenges of an aging population and the growing demand by the elderly population for health services, an increasing number of countries and regions are implementing telemedicine technology, which employs new-generation information technology to address the health problems of the elderly and promote the development of healthy aging in a local context. Major organizations and countries in the world have put forward corresponding policies to support the development of telemedicine technology in order to improve the current low popularity and utilization rate of telemedicine. For example, the Chinese government has put forward the Outline of the Healthy China 2030 Plan, which lists telemedicine and promoting the health of the elderly as important goals. The US Department of Health and Human Services released the 2023–2028 Data Strategy, which proposes the use of artificial intelligence to enhance the availability and equity of healthcare services. The British government put forward the Future Health Development of the UK plan, using the new generation of information technology to achieve precision medicine and disease prevention.

In China, adults over the age of 60 account for more than 21.1% of the population (about 296.97 million people), which has resulted in a significant increase in medical and healthcare demands [4]. In addition, the 52nd Statistical Report on the Development of the Internet in China shows that the proportion of people over 60 years old with Internet access exceeds 100 million [5]. In the past three years, in response to the COVID-19 epidemic, the Chinese government built a large number of Internet hospitals, home beds, telemedicine monitoring systems, and other such facilities. They introduced a large number of policies to support the use of telemedicine for the convenience of the elderly [6]. These changes make it much easier for older people to use telemedicine technology.

Telemedicine is the interchange of medical information from one site to another using electronic communication, to improve patients’ clinical health [7]. Telemedicine currently provides three categories of services. One is safety and security monitoring, which includes gas sensors and flood and fire detectors. The second category is monitoring health metrics and vital indicators like heart rate, blood pressure, temperature, and blood glucose levels. The third is assistance via information and communication technologies (Internet, telephony), such as teleconsultations, short message service (SMS) reminders for appointments and prescription drugs, and instructional text messages [8]. The promotion and application of telemedicine technologies can provide suitable solutions to improve the quality of care for older people, enable remote access, and reduce healthcare costs, thus promoting the empowerment of older people, improving monitoring, supporting home healthcare, and preventing chronic diseases [8].

The popularity of telemedicine among the older population is largely dependent on the basic telemedicine infrastructure (e.g., Internet availability, information and communication technology access, and telemedicine tools/software), as well as their level of adoption of this technology [9]. As mentioned above, in China, after the impact of the COVID-19 pandemic, a large amount of telemedicine infrastructure was built. This infrastructure makes it relatively easy to carry out telemedicine in China’s urban areas. The use of telemedicine can assist elderly people in accessing various forms of digital products, as well as facilitating their access to a greater choice of healthcare services [10]. Moreover, compared with other types of products (such as e-government products or mobile banking), using telemedicine involves interacting with more diverse people (such as caregivers, children, doctors, and service providers), which means that the elderly can gain intergenerational literacy in the process of using telemedicine and working with these people. Many information technology products involve only the use of a mobile phone. Telemedicine often involves the use of multiple products (such as the joint use of a mobile phone and electronic instrument), which provides an opportunity for the elderly to access other types of products. This will increase the likelihood that older people will expand their use of digital products in the future. For example, older persons who learn and master the use of telemedicine with the assistance of family members or healthcare practitioners may be more interested in using other types of digital products, increasing the range (breadth) and intensity (depth) of their digital product use. A high trust rate of patients for telemedicine in general will help telemedicine expand beyond the initial, expected users (i.e., younger patients, digital workers) and also increase the likelihood of use of other digital products, to increase the breadth and depth of digital technology use by older people [11].

Furthermore, the use of telemedicine technology not only allows older people to improve their health and well-being, and to more easily use other digital products, but also helps them bridge the digital gap, boost their digital social integration, and enjoy greater technical well-being. Due to the impact of factors like declining intellect, education, and age [12], older adults typically struggle to use digital gadgets and integrate into digital society. This means that this group of older adults loses their capacity to control their lives in the digital age, affecting their ability to enjoy the convenience and benefits offered by the digital society; examples are, the shrinking of older adults’ social networks, leading to anxiety and loneliness [13], the inability to independently make online purchases [14], and so on. But the application of telemedicine technology among the elderly population perfectly solves the series of problems that produces this “digital divide” [15]. As a result, a thorough understanding of the influence of telemedicine technology on older individuals’ sense of control over their digital lives is critical for fostering the advancement of this telemedicine technology and the ensuing benefits.

Just as the psycho-physio-social paradigm is widely employed in medicine, the research and development of telemedicine technology in geriatrics should be more thorough and widespread. However, prior research has focused on the problems in adopting telemedicine technology due to the digital divide, including but not limited to unstable Internet connectivity, lack of access to the Internet [16,17], restricted computer or smartphone access [18], ability of products to interact with customers [19], and so on. There is a paucity of research on the positive influence of telemedicine technology on bridging the digital divide and the ensuing positive effects, such as boosting cognitive function, self-care abilities, and the elderly’s sense of control over their lives. Especially in the current era of rapid development of telemedicine, research on technology alone cannot solve the difficulties of the elderly navigating the digital society and their subsequent problems with social integration and independent living. Telemedicine can improve the health of the elderly population while giving them the possibility of improving their social networks (meeting new nurses, doctors, service providers, etc.) and using more devices. Therefore, related research cannot be limited to only medical improvements and needs to include improvements in the quality of life. As a result, this study addresses the following questions to investigate the function of telemedicine in strengthening the elderly’s sense of control over their digital lives and alleviating the “digital divide phenomenon”.

How do older adults’ trust in, and expectations of, telemedicine technology influence the way they use digital technology?

How does older adults’ use of digital technology affect their sense of control over their digital lives?

In summary, the main research objective of this paper is to explore the mechanism by which use of remote medical services (telemedicine) affects the sense of control over digital life among the elderly. Through this study, we hope to fill the research gap on the usage behavior and post-use impact of remote medical services by the elderly and provide more in-depth and systematic validation results for the application of trust and expectation theory in regard to telemedicine. The main contribution of this study is to provide evidence for the development of trust theory and the prediction and expansion of telemedicine use among the elderly. Additionally, it provides theoretical support of how telemedicine expands the sense of control of the elderly, which is a new perspective on promoting the concept of life control in the digital era.

The rest of the study is organized as follows: Section 2 introduces the concept definitions and literature review of trust, expectations, and sense of control in digital life. In Section 3, the research hypotheses are proposed. Section 4 describes the scale used in the study and the method and process of data collection. Section 5 describes the process and results of the data analysis. In Section 6, the research results are discussed, and the contribution of this paper is described. Finally, we summarize the text and propose future research.

## 2. Literature Review

### 2.1. Trust

The concept of trust is diverse but can typically be defined as the willingness of one party to be susceptible to the behavior of another party based on the expectation that the other party will perform a specific act that is important to the one party, regardless of whether the one party has the ability to monitor or control the other party [20]. In the realm of telemedicine, patient trust in telemedicine services is defined as the patient’s willingness to rely on such health services (and the variables that compose this service) during treatment. In other words, trust is a patient’s readiness to put their health and privacy in the hands of a telemedicine service provider in exchange for personal benefits (such as enhanced quality of care or time savings). This trust is multidimensional, and it is most likely the sum of various components that compose a telemedicine service, each of which has an impact on the patient’s faith in telemedicine.

Given the diversity in the contexts, products, and service receivers, the factors that influence patient trust vary significantly among healthcare domains. For example, Velsen et al. classified trust variables affecting telemedicine as trust in the care provider, trust in the healthcare professional, and trust in the technology [21]. Marina et al. classified the bases of trust into four broad categories: the personality basis (positive beliefs in human nature and functionality of technological products and related services systems); the cognitive basis (cognitive familiarity, reputation of the technology, reliability of the technology and service providers, and human roles and structures); the calculative basis (risk of exposure to opportunistic behavior and positive beliefs in official advice); and the institutional basis (existence of “situational normality”, structural assurance, and control over personal information) [22]. Michler et al., on the other hand, categorized the influences on trust in smart products into users’ trust in the manufacturer and device, users’ assessment of the security of their personal data, and users’ perceived risk of the product [23].

For older people, trust can reduce the perceived risk and uncertainty associated with digital technologies, increase acceptance among older people, positively influence attitudes and behaviors toward use, and play a crucial role in the adoption of digital technologies and services, ensuring that the elderly receive satisfactory expectations and outcomes [24]. However, due to a variety of factors such as their education and access to information, older people have relatively little technical understanding of digital products, and the generation of their trust is more variable, as shown in one study [22]. Another study proved that patients’ concerns about potential information security and privacy issues can lead to mistrust of smart healthcare system technologies and vendors [25,26]. Older adults may have concerns about this technology because (1) they feel uncomfortable following the instructions of the intelligent assistive technology, (2) the technology does not support a perceived sense of trustworthiness, and (3) the technology is not a credible technological entity in its operation or existence [27].

Similarly, studies have revealed that 5G smart healthcare has severe security and privacy challenges, with a huge number of linked devices and sessions with unknown dangers during use [28], and that existing technology cannot adequately handle the security threats. Not only that, but product vendors that routinely collect, analyze, and retain user data [29] may generate worries among the elderly about the privacy and security of their personal information. To accept smart devices, older adults must trust both the producer and the device [30]. As a result, one may claim that security, technology, and suppliers all play an essential part in the trust of older people.

### 2.2. Expectations

Expectations are the benchmark by which patients measure the performance of the healthcare system during their visit. They are at the heart of patient happiness and the responsiveness of the healthcare system [31]. In terms of telemedicine, expectations are described as an individual’s anticipation or projection of future telemedicine service performance levels [32]. Existing research indicates that individual health expectations are influenced not only by elements connected to the health system, but also by the larger social, community, and familial milieu [33]. In addition, influencing factors related to healthcare expectations include socio-economic status, age, health status, and past experience interacting with the health system, among others [34]. As a result, significant differences exist in health expectations for groups with various characteristics.

Telemedicine could aid the older generation in many ways. For example, telemedicine based on the “Internet of things” can accurately identify the ideal period of availability of various devices and supplement their smooth and continuous operation, ensuring optimal patient usage and efficient scheduling of limited resources with services [35]. This use highlights a disease-prevention goal of telemedicine. In the elderly population with diabetes, the goal of telemedicine in the treatment of complex type I diabetes is to help patients better control their blood glucose levels by fine-tuning insulin dosage, whereas for type II diabetes, while treatment modifications may be needed, improvements in glycemic control will be based primarily on behavioral changes (reduced calorie and carbohydrate intake, increased physical activity) [36]. This use highlights telemedicine for the treatment of disease. In a third example, therapeutic exercise is frequently required in rehabilitation medicine to reduce symptoms in older persons with chronic pain. But social, transportation, and financial hurdles may make it difficult for patients to follow their exercise regimens, and they look for healthcare delivery models that better suit their preferences—such as using telerehabilitation at home to increase their compliance with their exercise therapy [37]. This use highlights a goal of obtaining general healthcare functions via telemedicine.

In summary, positive expectations for telemedicine can play a positive role in disease prevention, disease treatment, and healthcare for older people, by motivating the adoption and use of telemedicine by this population.

### 2.3. Sense of Control in Digital Life

The sense of control over one’s life, that is, the perception of one’s ability to influence one’s surroundings and obtain desired outcomes, has also been defined as a subjective expectation of one’s ability to exert influence over life circumstances and outcomes in one’s surroundings [38]. This sense of control is also known as control points, learned helplessness, control beliefs, and perceived control. It is influenced by age, race, gender, experience, and background factors such as education and marriage [39]. A sense of control over one’s life typically combines both sources of control and a sense of control. Existing research reveals that a higher sense of control over one’s life promotes increased autonomy, a reduced risk of chronic disease, better physiological functioning, improved psychological well-being, and a decreased chance of death [40]. Older people’s sense of control over their lives tends to decline with age, impacted by an increase in the quantity and severity of socially significant unpleasant experiences (for example, death of spouse, retirement, and fear of institutionalization). Furthermore, because of declining health conditions, including functional decline (e.g., the ability to do activities of daily living) and biological decline (e.g., immunological), older persons report a decreasing sense of control over their lives [41].

With the rapid development of the next generation of information technology, the increased level of digitization of human society provides new opportunities for older people to improve their sense of independence, social connectedness, and worthiness in situations where they are affected by declining health or the limited ability to function [42]. Smart dishwashers and cookers with remote controls, for example, can enable older persons with restricted mobility to carry out daily activities independently. Smartphones can also help older people increase their contributions to society, social support network, and cognitive capacities, as well as lower their levels of depression [43]. However, older people often lack a knowledge or understanding of many of the technologies and technology-related products available to them [44]. The use of digital products by older people is also hampered by physical conditions, attitudes, socio-economic factors, skill acquisition, and privacy and security concerns [45]. Many elderly individuals, for example, use common technologies such as ATMs or PIN payments and generally feel good about this use, yet they are unable to handle smartphones competently. Another example is automatic ticket machines, which are the most commonly used machinery in public transportation but are often difficult for older people to figure out [46]. During the 2019 COVID-19 outbreak, most older people preferred to make their appointments online because of isolation restrictions, and they were extremely satisfied with many aspects of these online appointments, such as the convenience, the time savings, the precise dosages, and the privacy protections in use [47]. However, the difficulty in operating technological equipment and the intricacy of the consultation procedure make it impossible for them to obtain this healthcare service.

Essentially, elderly individuals may struggle to efficiently use digital technology to fulfill their daily requirements. Consequently, they may experience a loss of control over their lives in the digital era, becoming dependent on external entities such as family, friends, or assisted living facilities, and impeding their ability to live independently and autonomously. The perception of digital control significantly influences the independent and autonomous existence of older individuals in the digital era.

## 3. Model Construction and Hypothesis Development

### 3.1. Expectations and Telemedicine Usage

The goals and expectations of older adults exert a substantial influence on their behavior. Expectations have a crucial role in shaping people’s behaviors toward telemedicine systems. Specifically, expectations are the primary motivators for older patients to use telemedicine technology. In the future, telemedicine will be extensively used to efficiently monitor the majority of patients from a distance and to identify, diagnose, prevent, and cure their severe medical conditions [48].

Individuals who have a desire to prevent disease are more inclined to use various functions of the telemedicine system to prevent the development or advancement of illnesses [49]. Individuals with expectations about how telemedicine can help them with their healthcare in general will need to prioritize preserving both their physical and mental health. Additionally, the necessity of considering diverse views will also lead to the use of more telemedicine functions.

The use of the telemedicine systems’ capabilities will increase among older persons who have several chronic or critical conditions that necessitate long-term treatment and rehabilitation. For instance, individuals who have experienced a mild to moderate stroke can use telerehabilitation features, including remote monitoring for patient assessment and clinical management, teletherapy, teleconsultation, telementoring, and tele-education for professionals and caregivers. These interventions aim to enhance the patient’s motor impairments [50]. For the management of chronic diseases, such as with elderly diabetic patients, telemedicine facilitates medical education and the dissemination of knowledge and technology, and telemonitoring involves the use of mobile devices or computers for the remote monitoring of the patient’s vital signs or indicators of the disease [44], which makes it easier for healthcare professionals to control medication intake according to the indicators [51].

For older persons with complicated health demands, the greater the number of expectations they have about how telemedicine can help them prevent and treat diseases and manage their general healthcare, the more they will use telemedicine solutions in various ways and forms. Thus, this study posits that the senior population proactively enhances the breadth and depth of their use of telemedicine because of their expectations of how it can help them with disease prevention, general healthcare management, and disease treatment.

The hypotheses are as follows:

**H1a.**  *Disease prevention expectations positively influence the breadth of telemedicine use among older adults.*

**H1b.**  *Healthcare expectations positively influence the breadth of telemedicine use among older adults.*

**H1c.**  *Disease treatment expectations positively influence the breadth of telemedicine use among older adults.*

**H2a.**  *Disease prevention expectations positively influence the depth of telemedicine use among older adults.*

**H2b.**  *Healthcare expectations positively influence the depth of telemedicine use among older adults.*

**H2c.**  *Disease treatment expectations positively influence the depth of telemedicine use among older adults.*

### 3.2. Trust and the Way Telemedicine Is Used

Multiple studies have consistently shown that establishing trust is a prerequisite for the effective use of different technologies. Furthermore, these studies have also confirmed that trust plays a significant and essential role for older persons [52]. Various aspects of trust can have a substantial impact on the use of telemedicine services by elderly individuals.

The level of confidence in the system’s security is determined by considerations such as personal privacy, economic risk, and other factors relevant to the group using the system. Trust in the system’s security is particularly important among older age groups, who are adversely impacted by a deficiency in digital literacy. The potential threat of compromising one’s personal privacy alone is sufficient to undermine the inclination to use a telemedicine system, before the risk of tangible financial harm resulting from the exposure of private data is even considered. This frequently results in diminished or hesitant adoption of telemedicine technologies among older individuals [53]. Conversely, when a highly secure technology is implemented in telemedicine and older individuals are educated about its security measures, they are more inclined to use it. Consequently, they broaden their usage by adopting different types of software or devices and modify their usage behavior by increasing the functionalities they use or the hours they spend using it [54,55]. Essentially, older individuals can enhance their use of a telemedicine system by expanding both the range (breadth) and intensity (depth) of their use, provided that the group possesses a sufficient level of confidence in the system’s security.

Trust in telemedicine technology has a crucial role in determining the adoption and usage of the system by older persons. Research has demonstrated that when a system product meets or exceeds performance expectations, it results in higher levels of satisfaction and trust. This, in turn, leads to an increased likelihood of users purchasing and using the system product [56]. Furthermore, users who have confidence and find telemedicine services beneficial also develop trust in the online environment of their healthcare institution, as well as in the Internet platform and service providers (i.e., doctors). This trust enhances their inclination to further use telemedicine services, thereby contributing to the extent and intensity of their telemedicine use [57].

Another form of trust that is crucial for telemedicine is faith in its service providers. Multiple empirical studies have shown that the likelihood of a customer continuing to use and recommend a product is often influenced by a higher level of trust in the product’s reliability and the platform it is offered on, as well as a higher level of trust in the service provider (such as a doctor or caretaker) [58,59]. The study conducted by Chau et al. further validates the idea that when customers trust an online provider, they tend to improve their engagement and broaden their use, such as by using the provider’s other items [60]. Therefore, we posit that the greater the level of trust users place in their service providers, the more they will use the services provided.

Overall, past research indicates that older individuals will actively broaden and deepen their use of telemedicine because of a heightened confidence or trust in the security, technology, and providers of the telemedicine system.

The assumptions are therefore as follows:

**H3a.**  *Security trust positively influences the breadth of telemedicine use among older adults.*

**H3b.**  *Technology trust positively influences the breadth of telemedicine use among older adults.*

**H3c.**  *Provider trust positively influences the breadth of telemedicine use among older adults.*

**H4a.**  *Security trust positively influences the depth of telemedicine use among older adults.*

**H4b.**  *Technology trust positively influences the depth of telemedicine use among older adults.*

**H4c.**  *Provider trust positively influences the depth of telemedicine use among older adults.*

### 3.3. Telemedicine Usage and Sense of Control in Digital Life

Telemedicine offers older individuals not just the prospect of enhanced health but also the opportunity to develop their digital literacy, gain greater control over their digital lives, and become more integrated into the digital community. According to the World Health Organization, telemedicine encompasses one of four parts, which involves the utilization of many forms of information and communication technology. Learning to use telemedicine technologies enables older individuals to access a wide range of digital equipment, which in turn enhances their quality of life and promotes their ability to live independently at home for longer periods of time, reducing the need for institutional care [61].

The user’s expectations of and confidence in the telemedicine system, along with the capability to use it independently and autonomously (after help and training from others, such as children, friends, and the community), show a mastery of both the theoretical knowledge and practical skills required to operate the telemedicine technology [62]. Regarding the increase and broadening of telemedicine use, elderly individuals will acquire knowledge of various digital technologies while using wearable devices. This knowledge helps develop their digital self-assurance, enhance their digital literacy, and subsequently improve their sense of mastery over their digital existence [63]. To summarize, elderly people will experience a heightened sense of autonomy in managing their digital life due to the availability of telemedicine services.

The assumptions are therefore as follows:

**H5a.**  *Breadth of telemedicine use positively affects digital process control sources.*

**H5b.**  *Depth of telemedicine use positively affects digital outcome control sources.*

**H6a.**  *Breadth of telemedicine use positively affects sense of control over digital processes*.

**H6b.**  *Depth of telemedicine use positively affects sense of control over digital outcomes.*

**Figure 1:**
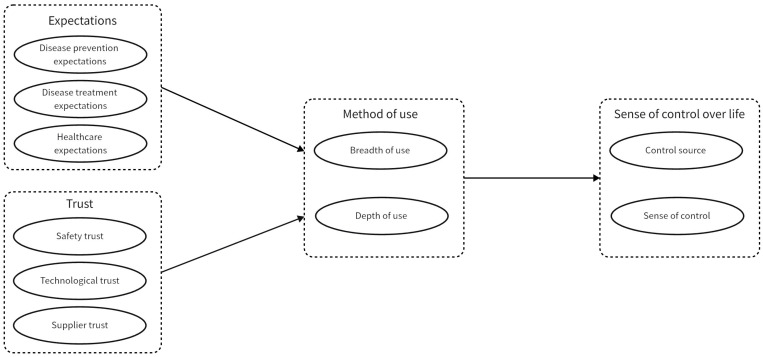
Research model.

The research model is shown in Figure 1.

## 4. Research Methodology

### 4.1. Measures

Drawing on the literature analysis and our hypotheses, we present a theoretical framework comprising four latent variables: expectations of telemedicine, trust in the telemedicine system, usage patterns for telemedicine, and the sense of control over digital life. More precisely, the measurement items for expectations of telemedicine were derived from the Treatment Expectations Questionnaire (TEX-Q) [64]; those for trust in telemedicine were adapted from Velsen, Tabak, and Hermens [21]; and those to measure telemedicine use patterns were from the Expanded Use and Integrated Use of Information Systems Scale, established by Kim [65], with the telemedicine system suitably modified to align with the characteristics of the scale.

The measurement items for sense of control over digital life consisted of two components: the source of control and the feeling of control. The source of control used a behavioral control point scale developed by Craig [66]. Behavior is often influenced by one’s sense of control, which can be measured using Schwarzer’s General Self-Efficacy Scale [67]; the Chinese iteration of the scale demonstrated commendable reliability and validity.

The measurement items were rated on a 7-point Likert scale, ranging from 1 (indicating strong disagreement) to 7 (indicating strong agreement). Table 1 lists the items.

### 4.2. Sample Selection and Data Collection

Data collection included two stages.

Initially, 50 pre-survey questionnaires were disseminated, and semi-structured interviews were carried out with the participants. Subsequently, the questionnaires were adjusted, taking into account the insights gained from the interviews, to guarantee their clarity and accuracy.

The formal questionnaire was conducted through both online and offline methods. All respondents were registered members of government-supported smart healthcare and smart elderly care service providers. These service providers are supported by the government to provide free or semi-free telemedicine services to the elderly. After obtaining approval from the relevant supervisory and competent authorities, questionnaires were issued. The online questionnaire was disseminated via the WeChat platform, and access protocols were implemented to ensure that any IP address or WeChat account could complete the questionnaire only once. Based on the business scope of the telemedicine service provider, the scope of the online questionnaire covered major regions and cities in China. The offline questionnaire was implemented using paper, and completion was facilitated by skilled community workers and students. Due to financial and capacity limitations, the offline questionnaire was distributed only in Anhui Province, China. After completing the questionnaire, respondents were given either cash of about US$1 or a gift of that value, depending on which gift they chose.

The questionnaires were gathered in April 2024 from a specific group of individuals who were 60 years of age or older and who used telemedicine systems or technology. Out of the total of 833 questionnaires gathered, 661 questionnaires were considered legitimate after removing the invalid ones. This resulted in a validity rate of 79.35%. Table 2 displays the attributes of the sample used in the questionnaire.

## 5. Data Analysis and Results

### 5.1. Measurement Model

This study employs partial least squares (PLS) for analysis. PLS is a more suitable method for conducting exploratory studies compared to other methods. Furthermore, PLS with structural equation modeling is capable of efficiently addressing the issue of uneven sample distribution due to its lenient criterion for data normality. Hence, the data analysis in this study was conducted using SmartPLS4 version 4.1.0.3 [68].

#### 5.1.1. Multicollinearity

The variance inflation factor (VIF) quantifies the extent of covariance in a multiple linear regression model. The VIF has a value that exceeds 1. A value approaching 1 indicates a lower probability of encountering a multicollinearity issue. The analysis found that the highest value of VIF is 4.707, which is below the threshold of 5. This suggests that the model does not exhibit multicollinearity.

#### 5.1.2. Confidence and Validity

Table 3 presents the factor loadings, Cronbach’s alpha, combined reliability (CR), and extracted mean variance (AVE). As per Hair’s (1998) suggestion, the factor loadings of the model should be a minimum of 0.60 and preferably 0.70 or above. The factor loadings of the model in this investigation varied between 0.703 and 0.859, surpassing the desired threshold of 0.7 [69]. This demonstrates the robust convergent validity of the observed variables and a significant link between the observed variables and their structural factors. CR is a crucial indicator of the internal consistency of the dimensions of a model and it should exceed 0.7 [69]. The CR values in this investigation range from 0.865 to 0.916, surpassing the cutoff value of 0.7. This suggests that the model demonstrates strong convergent validity. Cronbach’s alpha is a crucial indicator of the internal consistency of the model, and the majority of research indicates that it should exceed 0.7 [69]. The scores in this study vary from 0.765 to 0.900, surpassing the threshold value of 0.7. This indicates that the questionnaire exhibits strong internal consistency. Furthermore, the AVE values range from 0.523 to 0.680, all of which are above 0.5. This suggests that the observed term accounts for a significant portion of the variation compared to the error term, showing a high level of model aggregate validity [69].

Table 4 demonstrates discriminant validity, since the square root of all AVE values exceeds the specified thresholds. This indicates that the questionnaire questions possess strong discriminant validity [70]. Simultaneously, Hamid et al. regarded the heterotrait–monotrait ratio of correlations (HTMT) as a viable method for assessing discriminant validity, and hence we provide these findings. HTMT scores below 0.85 indicate discriminant validity [70,71]. As shown in Table 5, all HTMT values were less than 0.85.

### 5.2. Structural Model

In this study, we used SmartPLS4 for data analysis. Bootstrapping was adopted, and the maximum number of iterations was 5000. The specific results are shown in Table 6.

Finally, after adding control variables, this study analyzed the relationship between each variable. The test results show that age has no significant effect on the results of this model. In terms of education level, the group with the higher education level (high school education or above) showed a higher level of influence on the depth of use of disease treatment expectations than the group with the lower education level (middle school education or below). In the comparison of male and female participants, the male group showed a higher level of influence on the breadth of use on the control source than the female group. The details of this analysis are in the Supplementary Materials.

## 6. Discussion and Implications

### 6.1. Main Findings

This study assessed the effects of telemedicine technology on the senior population. By testing the hypotheses of the model we created, we confirmed 13 of the total 16 hypotheses, while the remaining 3 hypotheses were not supported. The findings were derived from the outcomes of the hypothesis testing.

(1) The influence of older adults’ expectations regarding their use of telemedicine devices on their actual usage behavior.

The analysis of expectations regarding the use of telemedicine devices among older adults revealed that disease prevention expectations (H1a, path coefficient = 0.092, T = 2.458, *p* = 0.014) and disease treatment expectations (H1c, path coefficient = 0.131, T = 3.007, *p* = 0.003) had a smaller impact on the extent of information technology device use compared to healthcare expectations (H1b, path coefficient = 0.599, T = 13.817, *p* < 0.001). This outcome may be attributed to the following factors: The treatment and prevention of diseases (such as diabetes and heart disease) primarily focus on certain fundamentals, so the effective management of these conditions necessitates the use of software or programs with specialized functionalities. By contrast, everyday healthcare necessitates a broader range of functions, including functions for daily nutrition, physical activity, and adequate rest and relaxation. One telemedicine solution or software is frequently insufficient to address the wide range of healthcare needs. This has led to an increase in the elderly population’s adoption of software to fulfill their daily healthcare needs.

This assertion is additionally supported by our findings related to health expectations and depth of use. In Hypothesis 2, the expectations for disease prevention (H2a) and disease treatment (H2c) were found to have a positive relationship with the depth of information technology device use. The path coefficients for disease prevention expectations and disease treatment expectations were 0.134 and 0.192, respectively. The corresponding T values were 2.748 and 2.934, and the *p* values were 0.006 and 0.003. However, the hypothesis regarding healthcare expectations and the depth of information technology device use (H2b) did not yield significant results. The path coefficient for this relationship was −0.033, with a T value of 0.692 and a *p* value of 0.489. This study revealed that older adults primarily use telemedicine products or programs to address specific health concerns, such as disease prevention or treatment. This requires a thorough understanding and proficient use of a particular telemedicine device or program to effectively manage the specific health issue. However, when healthcare needs are very extensive, a greater range of products is typically necessary to fulfill them. This is likely the primary reason why the impact of healthcare expectations on the extent of product usage is not significant. This discovery is significant as it may offer a theoretical explanation for how the health expectations of older persons influence their health habits. Furthermore, it offers fresh empirical data that support several unusual discoveries in previous research.

(2) The influence of trust in telemedicine devices on usage behavior in elderly individuals.

The analysis of the impact of trust in telemedicine equipment on the breadth of use among older adults revealed that trust in telemedicine product safety (H3a, path coefficient = 0.125, T = 2.548, *p* = 0.011) and technology (H3b, path coefficient = 0.187, T = 3.079, *p* = 0.002) had a significant positive effect on the breadth of use. Additionally, trust in the service provider (H3c, path coefficient = 0.284, T = 4.791, *p* < 0.001) was found to significantly increase the breadth of use of telemedicine products among older adults. This discovery validates prior research that indicates that older persons are more inclined to use a wider range of products when they trust the provider [72].

Older individuals obtain telemedicine products by three primary methods: family members purchase them, the government provides them with free distribution, and offline vendors market and sell them. Nevertheless, older individuals frequently encounter challenges in independently growing their use of these items or software, primarily due to their limited digital literacy and access to knowledge. To expand the scope of their use, they could get help from the younger demographic [73]; however, their offspring frequently do not have the time to assist older relatives [74]. As another option, the salesperson representing a vendor could help the elderly individuals during the product promotion and impart specific digital skills that would instill confidence in the elderly toward the vendor. This, in turn, would encourage them to use the product more frequently.

The analysis of the impact of trust in telemedicine equipment on the depth of use for older adults found that trust in security (H3a, path coefficient = 0.18, T = 5.097, *p* < 0.001) significantly increases the depth of use. By comparison, trust in telemedicine product providers (H3c, path coefficient = 0.01, T = 0.25, *p* = 0.802) and trust in technology (H4b, path coefficient = −0.021, T = 0.521, *p* = 0.603) did not have a significant impact on the depth of use. This study’s results further validate the conclusions of prior research, which indicate that the elderly demographic highly values personal privacy and property protection. The majority of research has indicated that older persons prioritize privacy and property security as their primary concerns when using new technological products. Furthermore, their usage of a product or program is frequently influenced by their views of security [75].

Additionally, older age groups are sometimes hindered by a lack of digital literacy, leading to an inherent resistance toward adopting new technologies. This resistance poses a challenge in promoting new technologies or products among older individuals [76]. Even when a user trusts a product supplier, older users typically just accept a new product for a certain feature or technology and do not have strong motivations to explore its full potential. This study is significant as it offers fresh empirical evidence for the advancement of trust theories and the challenges faced in the spread of information technology among older individuals.

(3) The influence of older adults’ telemedicine usage behavior on their perception of control over their digital lives.

The study’s findings indicate that the breadth of older people’s use of telemedicine devices or software has a stronger impact on their sense of control over digital life and their source of control, compared to the effect of depth of use. The path coefficients for the breadth of use (H5a) were 0.395 and 0.396, with corresponding T values of 8.102 and 0.396, both highly significant (*p* < 0.001). By contrast, the path coefficients for the depth of use (H5b) were 0.213 and 0.299, with corresponding T values of 4.188 and 6.176, also highly significant (*p* < 0.001). This illustrates the efficacy of the increased use of diverse telemedicine equipment or software among elderly individuals in terms of enhancing their autonomy in the digital realm.

The potential causes for this efficacy are as follows. The functions or capacities of different types of devices or software vary. This variety requires a comprehension of the digital society and the associated expertise to use it. When extensively used by elderly individuals, these technologies enable them to develop adequate digital literacy and digital thinking skills. By acquiring this knowledge and competence, older adults can gain a deeper understanding of the underlying principles of digital life. This, in turn, enhances their capacity to navigate and use various digital products in their everyday activities, ultimately empowering them to exert greater control over their digital existence.

The results of this investigation hold considerable importance. First, it broadens the existing theory on the telemedicine usage behavior of older individuals and offers literature-based evidence to support the prediction and expansion of their usage behavior. Furthermore, it offers additional theoretical backing to expand the perception of control over life among older individuals. This can offer fresh perspectives for advancing the notion of life control in the digital age.

### 6.2. Implications

This study offers the following practical contributions.

First, the influence of older persons’ expectations regarding telemedicine technology on their use behavior indicates that various health-related assumptions among this demographic have an impact on their actual usage patterns. In other words, expectations related to specific diseases and expectations related to general healthcare can produce different behaviors. Thus, during the design phase of telemedicine products, upgrading a product should include more than just ensuring its usability for older persons. Telemedicine systems should be created that are directed or universalized according to the intended goal of the product. Such systems will enhance the proficiency of older individuals in using a certain product comprehensively, while also enhancing their capacity to transition to a similar type of product. In addition, older individuals frequently have comorbidities, resulting in a diversity of functional demands associated with various disorders. Hence, it is crucial to take into account the transferability of activities and systems across different products while building a product range.

Furthermore, this study’s findings on trust in telemedicine technology indicate that the influence of various forms of trust is equally diverse. The level of trust in security directly influences the extent to which older individuals use a product and their subsequent behavior regarding its use. Enhancing and bolstering the confidence of older adults in product security is crucial for fostering and enhancing the adoption of telemedicine products and services within this demographic. Hence, service and product providers must augment and refine their communication regarding the safety of product usage to furnish dependable proof of this safety to older adults, as well as to their families, as the procurement and use of telemedicine products by older adults is frequently influenced by their children. Furthermore, the security of older adults’ information and property can be enhanced by relevant governmental agencies through heightened supervision and efficient disclosure.

Finally, the findings indicate that older adults need extensive exposure to digital products to develop adequate digital literacy and competence, which in turn enhances their sense of control over their digital lives. Hence, apart from the requirement for telemedicine service providers to prioritize the guidance and adaptability of the product system, other groups (such as the children of elderly individuals, telemedicine advocates, community workers, and physicians) must support older adults as they learn to use telemedicine products or services. Crucially, others must actively assist them in adopting a digital mindset and offer the necessary support to address the challenges they encounter in their daily routines.

## 7. Conclusions and Limitations

This study investigates the influence of telemedicine product use on the sense of control that older persons have over their digital lives. It uncovers the processes via which telemedicine products can enhance the daily life capabilities of older adults. The study revealed the following findings: (1) Older adults are more likely to use a wider range of telemedicine products if they have high expectations for how the products can help with healthcare expectations. (2) Trust in the safety of telemedicine products leads to a greater extent of product use among older adults. (3) Older adults are more likely to use a wider range of telemedicine products if they trust the service provider. (4) The breadth of telemedicine product use has a stronger impact on older adults’ sense of control over their digital lives, compared to the depth of product use. The findings offer fresh empirical data supporting hypotheses from previous research concerning the role of expectations, trust, and sense of life control. In addition, they offer tangible contributions toward enhancing the use of telemedicine products among older individuals, promoting their digital literacy and proficiency, and enhancing their sense of agency in their digital existence.

This study presents significant findings but is also limited by some research deficiencies. This study, being cross-sectional in nature, did not have time-series-based information to support the relevant findings over a period of time. In particular, due to the impact of the COVID-19 pandemic, medical technology has been rapidly developing, which has made the use of telemedicine significantly different in different time periods. Therefore, longitudinal studies should be conducted to discover the differences and the impact of the COVID-19 pandemic. Furthermore, cultural and urban infrastructure differences contribute to the variation in health expectations and trust among older persons across different cultures. Therefore, the conclusions of this paper may not be universal. Future research needs to further collect regional data from different cultures and different infrastructure levels for comparative analysis. Finally, this study employed a questionnaire survey for data collection. Such a survey could have social consensus bias, which could introduce bias into the study’s findings. Hence, in the future, a comparative study of various data collection methods (such as interviews, data crawling, etc.) could enhance the reliability of the findings.

## Figures and Tables

**Table 1:** Variables and indicators.

Latent Variable		Measurement Item	Reference
Expectations	Disease Prevention Expectations	DPE1: I’m hoping the telemedicine system will slow down the disease.	[64]
		DPE2: I hope the telemedicine system will reduce the likelihood of abnormal physiological indicators.	
		DPE3: I want the telemedicine system to maintain my quality of life.	
	Healthcare Expectations	HE1: I want the telemedicine system to improve my quality of life.	
		HE2: I would like the telemedicine system to help me with the symptoms (of my illness).	
		HE3: I want the telemedicine system to improve my daily activities.	
	Disease Treatment Expectations	DTE1: I want the telemedicine system to improve my health.	
		DTE2: I want the telemedicine system to improve my outcomes.	
		DTE3: I want the telemedicine system to reduce my pain.	
Trust	Safety Trust	SAT1: Telemedicine systems can be trusted and keep my health information secure.	[21]
		SAT2: Telemedicine systems can be trusted and keep my private information secure.	
		SAT3: Telemedicine systems can be trusted and keep my treatment information secure.	
	Technological Trust	TT1: Telemedicine systems have a good level of information technology.	
		TT2: Telemedicine systems have a good level of medical technology.	
		TT3: Telemedicine systems have good system quality.	
	Supplier Trust	SUT1: Vendors of telemedicine systems have good reputations.	
		SUT2: Vendors of telemedicine systems can be socially responsible.	
		SUT3: Trusted after-sales service is available from telemedicine system providers.	
Usage	Depth of Use	DOU1: I use most of the available telehealth system features to support my health management.	[65]
		DOU2: I use all available telemedicine systems to help me perform health management.	
		DOU3: I take full advantage of the available telemedicine system features to complete my health management.	
	Breadth of Use	BOU1: I use the telemedicine system to better relate to the telemedicine system mission.	
		BOU2: I use the telemedicine system to link related telemedicine system tasks.	
		BOU3: I use a telemedicine system to coordinate multiple health management tasks.	
Sense of Control in Digital Life	Control Source	CS1: In a digital society, I’m confident I can handle the future.	[66]
		CS2: In a digital society, I believe a person can be the master of their destiny.	
		CS3: In a digital society, I think humans are victims of circumstances beyond their control.	
		CS4: In a digital society, my life is controlled by the outside world.	
	Sense of Control	SOC1: In a digital society, if I do my best, I’ve always been able to solve problems.	[67]
		SOC2: In a digital society, even if people are against me, I can still get what I want.	
		SOC3: In a digital society, it’s easy for me to stick to my vision and reach my goals.	
		SOC4: If I put in the necessary effort, I can certainly solve most of the puzzles life gives me.	
		SOC5: I can face difficulties calmly because I trust my ability to deal with them.	
		SOC6: When faced with a difficult problem, I can usually find several solutions.	

**Table 2:** Sample characteristics.

	Measure	Item	Count
Sex	Male	296	44.78%
	Female	365	55.22%
Education	Primary and below	68	10.29%
	Junior high school	232	35.10%
	Senior high school	217	32.83%
	University and above	144	21.79%
Age	60–64	242	36.61%
	65–69	166	25.11%
	70–74	120	18.15%
	75–79	96	14.52%
	80 and above	37	5.60%

**Table 3:** Reliability and validity.

Construct	Item	Loading	Cronbach’s α	CR	AVE
Sense of control (SOC)	SOC1	0.866	0.946	0.957	0.786
	SOC2	0.891			
	SOC3	0.880			
	SOC4	0.915			
	SOC5	0.884			
	SOC6	0.884			
Breadth of use (BOU)	BOU1	0.900	0.907	0.942	0.844
	BOU2	0.941			
	BOU3	0.914			
Disease treatment expectations (DTE)	DTE1	0.929	0.923	0.951	0.866
	DTE2	0.935			
	DTE3	0.927			
Healthcare expectations (HE)	HE1	0.932	0.93	0.956	0.877
	HE2	0.929			
	HE3	0.949			
Depth of use (DOU)	DOU1	0.939	0.926	0.953	0.871
	DOU2	0.938			
	DOU3	0.923			
Control source (CS)	CS 1	0.894	0.898	0.929	0.766
	CS 2	0.905			
	CS 3	0.868			
	CS 4	0.832			
Technological trust (TT)	TT1	0.939	0.936	0.959	0.886
	TT2	0.947			
	TT3	0.937			
Safety trust (SAT)	SAT1	0.885	0.864	0.917	0.786
	SAT2	0.902			
	SAT3	0.873			
Disease prevention expectations (DPE)	DPE1	0.869	0.881	0.927	0.808
	DPE2	0.934			
	DPE3	0.892			
Supplier trust (SUT)	SUT1	0.945	0.94	0.962	0.893
	SUT2	0.951			
	SUT3	0.939			

**Table 4:** Results of the discriminant validity analysis.

	BOU	DOU	SUT	HE	SAT	TT	SOC	CS	DTE	DPE
BOU	0.918									
DOU	0.530	0.933								
SUT	0.697	0.564	0.945							
HE	0.490	0.851	0.538	0.937						
SAT	0.488	0.745	0.491	0.771	0.887					
TT	0.670	0.557	0.741	0.543	0.490	0.941				
SOC	0.509	0.423	0.564	0.44	0.447	0.540	0.887			
CS	0.555	0.509	0.626	0.504	0.454	0.606	0.658	0.875		
DTE	0.672	0.568	0.770	0.518	0.453	0.777	0.535	0.598	0.931	
DPE	0.582	0.586	0.625	0.550	0.528	0.600	0.501	0.518	0.578	0.899

Notes: DPE = Disease Prevention Expectations; HE = Healthcare Expectations; DTE = Disease Treatment Expectations; SAT = Safety Trust; TT = Technological Trust; SUT = Supplier Trust; DOU = Depth of Use; BOU = Breadth of Use; CS = Control Source; SOC = Sense of Control.

**Table 5:** HTMT analysis.

	BOU	DOU	SUT	HE	SAT	TT	SOC	CS	DTE	DPE
BOU										
DOU	0.578									
SUT	0.754	0.605								
HE	0.534	0.846	0.576							
SAT	0.551	0.832	0.543	0.838						
TT	0.727	0.599	0.790	0.582	0.544					
SOC	0.546	0.451	0.595	0.469	0.496	0.571				
CS	0.614	0.557	0.681	0.552	0.516	0.661	0.715			
DTE	0.734	0.615	0.826	0.559	0.507	0.836	0.570	0.656		
DPE	0.651	0.649	0.687	0.607	0.604	0.661	0.546	0.582	0.641	

Notes: DPE = Disease Prevention Expectations; HE = Healthcare Expectations; DTE = Disease Treatment Expectations; SAT = Safety Trust; TT = Technological Trust; SUT = Supplier Trust; DOU = Depth of Use; BOU = Breadth of Use; CS = Control Source; SOC = Sense of Control.

**Table 6:** Hypothesis testing.

	Hypothetical Path	Path Coefficient	T Value	*p* Values	
H1a	DPE -> BOU	0.092	2.458	0.014	Support
H1b	HE -> BOU	0.599	13.817	<0.001	Support (1)
H1c	DTE -> BOU	0.131	3.007	0.003	Support
H2a	DPE -> DOU	0.134	2.748	0.006	Support
H2b	HE -> DOU	–0.033	0.692	0.489	Unsupported
H2c	DTE -> DOU	0.192	2.934	0.003	Support
H3a	SAT -> BOU	0.125	2.548	0.011	Support
H3b	TT -> BOU	0.187	3.079	0.002	Support
H3c	SUT -> BOU	0.284	4.791	<0.001	Support (3)
H4a	SAT -> DOU	0.180	5.097	<0.001	Support (2)
H4b	TT -> DOU	–0.021	0.521	0.603	Unsupported
H4c	SUT -> DOU	0.010	0.250	0.802	Unsupported
H5a	BOU -> SOC	0.395	8.102	<0.001	Support
H5b	DOU -> SOC	0.213	4.188	<0.001	Support
H6a	BOU -> CS	0.396	7.800	<0.001	Support
H6b	DOU -> CS	0.299	6.176	<0.001	Support (4)

Notes: DPE = Disease Prevention Expectations; HE = Healthcare Expectations; DTE = Disease Treatment Expectations; SAT = Safety Trust; TT = Technological Trust; SUT = Supplier Trust; DOU = Depth of Use; BOU = Breadth of Use; CS = Control Source; SOC = Sense of Control. (1) Healthcare expectations promote the breadth of telemedicine product use; (2) trust in product safety increases the depth of telemedicine product use; (3) trust in the service provider promotes the breadth of telemedicine product use; and (4) when compared to the depth of product use, the breadth of telemedicine product use increases older adults’ sense of control over their digital lives.

## Data Availability

The raw data supporting the conclusions of this article will be made available by the authors on request.

## Supplementary Materials

The following supporting information can be downloaded at: https://www.mdpi.com/article/10.3390/healthcare12171685/s1, Table S1. Age group differences (60–70 vs 70+). Table S2. Gender differences. Table S3. Differences in educational attainment (middle school and below VS high school and above).
