# Supplementary material for: How Expectations and Trust in Telemedicine Contribute to Older Adults’ Sense of Control: An Empirical Study
Source: Healthcare (Basel). 2024 Aug 23;12(17):1685. doi: 10.3390/healthcare12171685 (PMC11394832; doi:10.3390/healthcare12171685)
Supplement: Supplementary file 1 [file healthcare-12-01685-s001.zip › healthcare-3136532-supplementary.pdf]

Table S1. Age group differences (60–70 vs 70+).

	Difference(Age (low) - Age (high))	1-tailed(Age (low) vs Age (high))	2-tailed (Age (low) vs Age (high))
Breadth of Use -> Sense of Control	0.096	0.157	0.315
Breadth of Use -> Control Source	0.032	0.377	0.754
Depth of Use -> Sense of Control	0.025	0.404	0.809
Depth of Use -> Control Source	0.074	0.228	0.456
Supplier Trust -> Breadth of Use	0.027	0.414	0.827
Supplier Trust -> Depth of Use	-0.008	0.541	0.918
Healthcare Expectations -> Breadth of Use	0.132	0.09	0.181
Healthcare Expectations -> Depth of Use	-0.025	0.61	0.78
Safety Trust -> Breadth of Use	-0.051	0.695	0.609
Safety Trust -> Depth of Use	-0.083	0.873	0.254
Technological Trust -> Breadth of Use	0.024	0.421	0.842
Technological Trust -> Breadth of Use	0.103	0.1	0.2
Disease Treatment Expectations -> Breadth of Use	0.01	0.473	0.946
Disease Treatment Expectations -> Depth of Use	-0.007	0.529	0.941
Disease Prevention Expectations -> Breadth of Use	-0.083	0.821	0.358
Disease Prevention Expectations -> Depth of Use	0.024	0.383	0.766

Table S2. Gender differences.

	Difference (Gender female. - Gender male)	1-tailed (Gender female vs gender male) p value	2-tailed (Gender female vs gender male p value
Breadth of Use -> Sense of Control	0.039	0.336	0.673
Breadth of Use -> Control Source	0.197	0.022	**0.044**
Depth of Use -> Sense of Control	-0.094	0.791	0.417
Depth of Use -> Control Source	-0.066	0.72	0.559
Supplier Trust -> Breadth of Use	0.052	0.334	0.667
Supplier Trust -> Depth of Use	0.099	0.142	0.285
Healthcare Expectations -> Breadth of Use	-0.028	0.593	0.813
Healthcare Expectations -> Depth of Use	-0.121	0.874	0.252
Safety Trust -> Breadth of Use	-0.108	0.834	0.333
Safety Trust -> Depth of Use	0.13	0.066	0.132
Technological Trust -> Breadth of Use	0.118	0.165	0.331
Technological Trust -> Breadth of Use	-0.012	0.545	0.91
Disease Treatment Expectations -> Breadth of Use	-0.122	0.775	0.45
Disease Treatment Expectations -> Depth of Use	-0.014	0.555	0.89
Disease Prevention Expectations -> Breadth of Use	0.143	0.086	0.171
Disease Prevention Expectations -> Depth of Use	-0.095	0.874	0.252

Table S3. Differences in educational attainment (middle school and below VS high school and above).

	Difference (Education Level (High) - Education Level (Low))	1-tailed (Education level (high) vs Education level (low)) p value	2-tailed (Education level (high) vs Education level (low)) p value
Breadth of Use -> Sense of Control	-0.134	0.914	0.172
Breadth of Use -> Control Source	0.015	0.445	0.89
Depth of Use -> Sense of Control	0.006	0.476	0.952
Depth of Use -> Control Source	-0.101	0.845	0.309
Supplier Trust -> Breadth of Use	-0.073	0.739	0.522
Supplier Trust -> Depth of Use	0.092	0.134	0.269
Healthcare Expectations -> Breadth of Use	-0.047	0.692	0.617
Healthcare Expectations -> Depth of Use	-0.119	0.92	0.16
Safety Trust -> Breadth of Use	0.143	0.056	0.112
Safety Trust -> Depth of Use	0.004	0.475	0.95
Technological Trust -> Breadth of Use	0.081	0.242	0.483
Technological Trust -> Breadth of Use	-0.09	0.88	0.239
Disease Treatment Expectations -> Breadth of Use	-0.201	0.944	0.112
Disease Treatment Expectations -> Depth of Use	0.191	0.012	**0.025**
Disease Prevention Expectations -> Breadth of Use	0.022	0.398	0.795
Disease Prevention Expectations -> Depth of Use	-0.03	0.661	0.678
